# Fatty Acids Rescue the Thermogenic Function of Sympathetically Denervated Brown Fat

**DOI:** 10.3390/biom11101428

**Published:** 2021-09-29

**Authors:** Qiang Cao, Shirong Wang, Huan Wang, Xin Cui, Jia Jing, Liqing Yu, Hang Shi, Bingzhong Xue

**Affiliations:** 1 Department of Biology, Georgia State University, Atlanta, GA 30303, USA; caoqiang78@gmail.com (Q.C.); swang38@student.gsu.edu (S.W.); xcui@gsu.edu (X.C.); jjing@gsu.edu (J.J.); 2 Division of Endocrinology, Diabetes and Nutrition, Department of Medicine, University of Maryland School of Medicine, Baltimore, MD 21201, USA; jiujiu0427@126.com

**Keywords:** sympathetic nerve system, brown fat, thermogenesis

## Abstract

Sympathetic nervous system (SNS) innervation into brown adipose tissue (BAT) has been viewed as an impetus for brown fat thermogenesis. However, we surprisingly discovered that BAT SNS innervation is dispensable for mice to maintain proper body temperature during a prolonged cold exposure. Here we aimed to uncover the physiological factors compensating for maintaining brown fat thermogenesis in the absence of BAT innervation. After an initial decline of body temperature during cold exposure, mice with SNS surgical denervation in interscapular BAT gradually recovered their temperature comparable to that of sham-operated mice. The surgically denervated BAT also maintained a sizable uncoupling protein 1 (UCP1) protein along with basal norepinephrine (NE) at a similar level to that of sham controls, which were associated with increased circulating NE. Furthermore, the denervated mice exhibited increased free fatty acid levels in circulation. Indeed, surgical denervation of mice with CGI-58 deletion in adipocytes, a model lacking lipolytic capacity to release fatty acids from WAT, dramatically reduced BAT UCP1 protein and rendered the mice susceptible to cold. We conclude that circulating fatty acids and NE may serve as key factors for maintaining BAT thermogenic function and body temperature in the absence of BAT sympathetic innervation.

## 1. Introduction

Obesity poses a serious risk for the development of a panel of metabolic diseases that greatly increase morbidity and mortality in current society [1]. Obesity is a state of chronic energy imbalance resulting from caloric intake greater than energy expenditure [1]. While white adipose tissue (WAT) stores excess energy in the form of triglyceride, brown adipose tissue (BAT) dissipates energy through adaptive thermogenesis via UCP1, a mitochondrial inner membrane protein that uncouples oxidative phosphorylation from ATP synthesis to generate heat, thereby profoundly promoting energy expenditure [2]. There exist two kinds of thermogenic adipocytes. One is the classic brown fat that is confined to anatomically defined regions such as interscapular BAT (iBAT) and the other are inducible beige adipocytes that reside sporadically within WAT and can be induced by cold, β-adrenergic agonists, PPARγ agonists, myokines (e.g., irisin) and hepatokines (e.g., FGF21) [2,3,4,5,6]. Emerging evidence supports that brown/beige fat can also perform adaptive thermogenesis through UCP1-independent mechanisms [7,8,9,10]. Given the recent insights into the functional BAT in humans [11,12,13], activation of brown/beige adipocyte thermogenesis represents a promising strategy for therapeutic treatment of obesity.

The sympathetic nervous system (SNS) has been recognized as a key impetus for promoting BAT thermogenesis and WAT lipolysis through direct innervation into these tissues [14,15,16,17,18]. We also demonstrated that SNS innervation in WAT is required for beige adipocyte induction during a cold challenge [19]. However, hamsters with bilateral chemical denervation of sympathetic nerves in iBAT can tolerate an overnight cold challenge at 4 °C [20]. Similarly, mice with bilateral iBAT chemical SNS denervation can tolerate a seven-day cold challenge and maintain a significant amount of UCP1 protein in iBAT [19]. Of note, these animals exhibited increased beige adipocyte formation, which could compensate for the impaired iBAT function with ablated SNS innervation [19,20]. However, in these studies, SNS denervation in iBAT was achieved by microinjection of 6-hydroxydopamine (6-OHDA), a selective neurotoxin to SNS nerves, which led to a partial SNS denervation [19,20]. It is not clear whether the residue sympathetic innervation is responsible for the preservation of BAT thermogenesis to a certain degree that is sufficient to maintain body temperature. Furthermore, it remained unclear whether there are factors that could contribute to the maintenance of brown fat thermogenesis and body temperature in the absence of BAT SNS innervation.

Thus, in the present study, we applied a surgical denervation approach to completely abolish SNS innervation in iBAT. We found that mice with bilateral iBAT SNS denervation can tolerate a seven-day cold challenge at 5 °C. After an initial decline of body temperature in the first 24 h of cold challenge, these surgically denervated mice gradually recovered their body temperature to the level comparable to that of control mice, with only a slightly decreased UCP1 protein level in the denervated iBAT. We further explored the factors that are responsible for maintaining the cold tolerance and UCP1 protein levels in iBAT in mice in the absence of iBAT SNS innervation.

## 2. Materials and Methods

### 2.1. Mice

All procedures conducted in the animal studies were approved by the Institutional Animal Care and Use Committee at Georgia State University (GSU) (Animal protocol A19003). Male C57BL/6J mice used for all experiments were purchased from Jackson Laboratory (Bar Harbor, ME, USA). Mice with CGI-58 knockout in adipocytes (AC58KO mice Atlanta, GA, USA) were generated by crossing Comparative Gene Identification-58 (CGI58)-floxed mice [21] with Adiponectin-Cre mice (Jackson Laboratory, Stock No 028020 Bar Harbor, ME, USA), where Cre is specifically expressed in mature adipocytes including white and brown adipocytes. Mice were housed in a temperature- and humidity-controlled animal facility on a 12/12 h light/dark cycle and had ad libitum access to water and a regular chow diet (catalog no.: 5001; LabDiet; Purina, St. Louis, MO, USA). At the end of the studies, mice were euthanized and all WAT and BAT depots were dissected, weighed, snap-frozen in liquid nitrogen, and stored at −80 °C for gene and protein expression analysis or fixed in 10% neutral formalin for immunohistochemical analysis.

### 2.2. Surgical Denervation of SNS in Interscapular BAT (iBAT)

12-week-old male C57/BL6J mice were randomly divided into two groups: one for surgical denervation and the other for sham surgery. iBAT SNS denervation was achieved by surgically cutting 0.5cm–1.0cm intercostal nerves (5 total nerves) bilaterally. Mice with surgical or sham denervation went on 7-day recovery and then were either housed at room temperature as control groups or challenged with cold at 5 °C for 16 h or 7 days.

### 2.3. Cold Exposure

Mice with sham or surgical denervation were subjected to a cold challenge (5 °C) in a time course from 16 h up to seven days. At the end of the experiment, adipose tissues were dissected, weighed, snap-frozen in liquid nitrogen and stored for further analysis of protein expression and immunohistochemistry. In some cold exposure experiments, a temperature transponder (BioMedic Data Systems, Seaford, DE, USA) was implanted into the mouse peritoneal cavity to monitor the body temperature, as previously described [20]. For AC58KO mice, food was removed 4 h before the initiation of cold exposure and remained absent during the cold exposure. For gavage experiments, food was removed 4 h before the cold exposure and followed by olive oil gavage 30 min before the cold exposure.

### 2.4. Immunoblotting

Adipose tissue was homogenized in a modified radioimmunoprecipitation assay (RIPA) lysis buffer supplemented with a protease inhibitor mixture and phosphatase inhibitor mixture (Sigma, St. Louis, MO, USA). Tissue homogenates were separated by SDS-PAGE, which were transferred to nitrocellulose membrane (Bio-Rad, Hercules, CA, USA), followed by blocking, washing, and incubating with primary antibodies and Alexa Fluor 680-conjugated secondary antibodies (ThermoFisher Scientific, Waltham, MA, USA). The blots were developed with a Li-COR Imager System (Li-COR Biosciences, Lincoln, NE, USA). The primary antibodies are UCP1 (1:500, Abcam, ab23841, Cambridge, MA, USA), TH (1:1000, Millipore, AB152, Burlington, MA, USA), phospho-HSL (pHSL) (1:1000, Cell Signaling, 4126, Danvers, MA, USA), CD36 (1:1000, Abnova, PAB 12463, Walnut, CA, USA) and α-Tubulin (1:1000, Advanced BioChemicals, ABCENT4777, Lawrenceville, GA, USA).

### 2.5. Immunohistochemistry

Fat tissue was fixed in 10% neutral formalin, embedded in paraffin, and cut into 5 µm sections, which were further processed for hematoxylin and eosin (H&E) staining or immunostaining with the UCP1 antibody (1:150, Abcam, ab10983, Cambridge, MA, USA), as previously described [19].

### 2.6. Norepinephrine (NE) Turnover and NE Content Measurement

Norepinephrine turnover (NETO) analysis was conducted using the a-methyl-p-tyrosine (AMPT) method as previously described [22]. Briefly, one cohort of mice were intraperitoneally injected with AMPT, a competitive inhibitor of tyrosine hydroxylase, twice with 2-h apart at doses of 300 and 150 mg/kg body weight, respectively, to inhibit tyrosine hydroxylase (TH). A second cohort of mice were sacrificed to obtain basal norepinephrine (NE) for subsequent calculation of NETO. All mice were euthanized 4 h later after the first administration of AMPT. All fat pads including BAT and WAT were dissected, weighed, snap-frozen in liquid nitrogen, and then stored at 80 °C freezer for further analysis of catecholamine contents. The NE contents in tissues were measured using reverse phase HPLC with electrochemical detection as previously described [22]. Briefly, fat tissue was homogenized in a solution containing dihydroxybenzylamine (DHBA, internal standard), perchloric acid (PCA) and ascorbic acid (AA). Catecholamines were extracted from the homogenate with alumina and were then eluted into PCA/AA. Catecholamine levels were measured using a HPLC system with electrochemical detection (Coulochem II). NETO was calculated in BAT and WAT, as previously described [22].

### 2.7. Serum Assays

Serum free fatty acids were quantified using an HR Series NEFA kit (Wako Diagnostics, Richmond, VA, USA). Serum catecholamines were quantified using an ELISA kit (E4462, Biovision, Milpitas CA, USA). Serum glucocorticoids were quantified using an ELISA kit (Ab108821, Abcam, Cambridge, MA, USA).

### 2.8. Statistical Analysis

All data are expressed as mean ± SEM. Statistical differences between groups were analyzed by student *t* test or one-way ANOVA as appropriate. Statistical significance is accepted at *p <* 0.05.

## 3. Results

**Figure 1:**
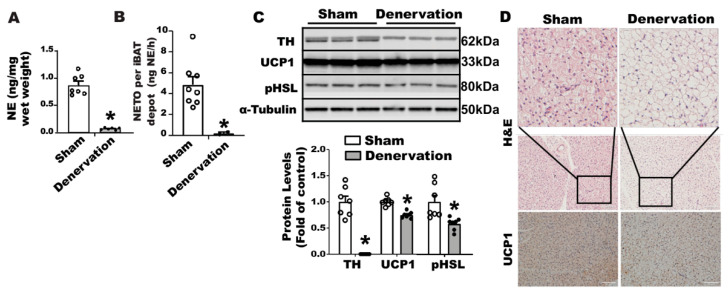
Surgical SNS denervation in iBAT reduces iBAT NETO and UCP1 levels in mice housed at room temperature. (**A**) NE content in iBAT of denervated and sham-operated mice. (**B**) NETO in iBAT of denervated and sham-operated mice. (**C**) Immunoblots of TH, phospho-HSL (pHSL) and UCP1 proteins in iBAT of denervated and sham-operated mice. (**D**) H&E staining and immunohistochemical staining of UCP1 in iBAT of denervated and sham-operated mice. Twelve-week-old C57BL/6J male mice were subjected to a surgical or sham bilateral SNS denervation in iBAT. All data are expressed as mean ± SEM; n = 6–8; * *p* < 0.05 vs. sham.

To determine the importance of SNS innervation in BAT thermogenic function and whole body thermoregulation, we conducted a bilateral surgical SNS denervation in interscapular BAT (iBAT). After a seven-day recovery from surgery at room temperature, the denervated mice were overall healthy and weighed similarly to sham-operated control mice (Supplementary Figure S1A). While the denervated iBAT appeared to be pale (Supplementary Figure S1B), there was no difference in fat pad weight including iBAT, inguinal WAT (iWAT), epididymal WAT (eWAT) and retroperitoneal WAT (rWAT) between the denervated and sham-operated mice (Supplementary Figure S1C). We next confirmed denervation efficacy neurochemically by assessing norepinephrine (NE) content and the norepinephrine turnover (NETO) rate in fat tissues. Surgical denervation significantly reduced both basal NE and NETO in iBAT of denervated mice compared to that of sham operated control mice (Figure 1A,B). A further characterization revealed no detectable tyrosine hydroxylase (TH) protein (Figure 1C) in denervated iBAT, confirming a complete sympathetic denervation. However, the denervated iBAT exhibited only slightly reduced UCP1 and phospho-HSL (pHSL) levels compared to that of sham-operated mice (Figure 1C). Immunohistochemical analysis also showed slightly larger brown adipocytes and slightly less UCP1 staining in denervated iBAT compared to those of sham controls (Figure 1D).

**Figure 2:**
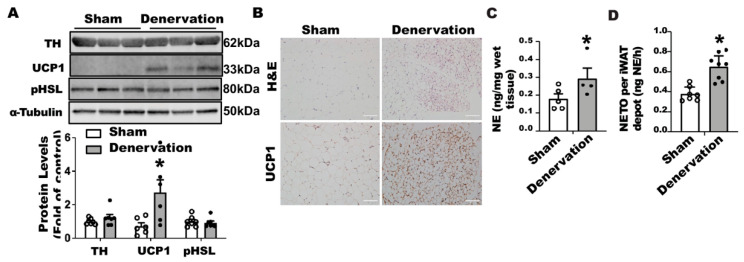
Surgical SNS denervation in iBAT increases NETO and beiging in iWAT of mice housed at room temperature. (**A**) Immunoblots of TH and UCP1 protein in iWAT of denervated and sham-operated mice. (**B**) H&E images and immunohistochemical staining of UCP1 in iWAT of denervated and sham-operated mice. (**C**) NE content in iWAT of denervated and sham-operated mice. (**D**) NETO in iWAT of denervated and sham-operated mice. Twelve-week-old C57BL/6J male mice were subjected to a surgical or sham bilateral SNS denervation in iBAT. All data are expressed as mean ± SEM; n = 4–8; * *p* < 0.05 vs. sham.

In contrast, iBAT SNS denervation markedly induced UCP1 protein contents in iWAT, with a tendency seen for increased TH protein but no change in phospho-HSL (Figure 2A). Immunohistochemical analysis further disclosed more UCP1-positive multilocular adipocytes in iWAT of the denervated mice (Figure 2B), suggesting an induction of beiging in these mice. Interestingly, iBAT SNS denervated mice exhibited an up-regulation of both basal NE and NETO in iWAT (Figure 2C,D), suggesting a compensatory up-regulation of sympathetic innervation in iWAT, which may contribute to enhanced beiging in iWAT.

**Figure 3:**
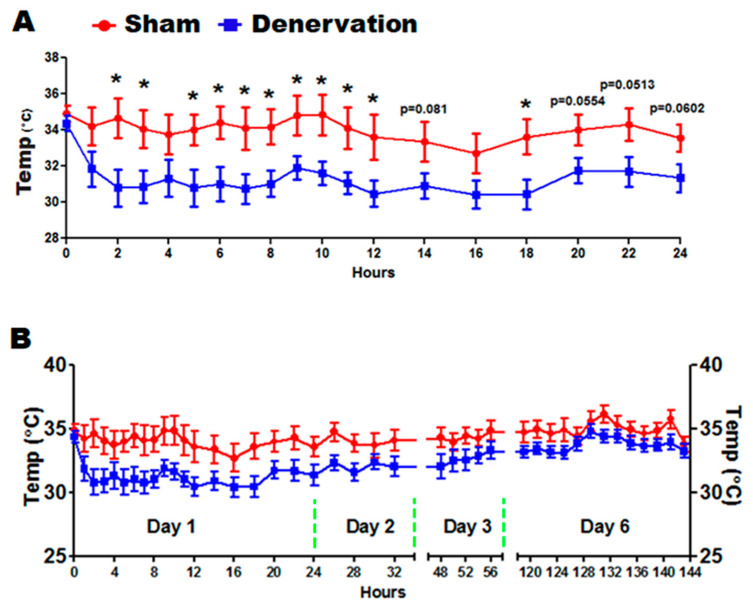
Body temperature of mice with iBAT surgical SNS denervation or sham operation during a seven-day cold exposure. (**A**) Body temperature in mice during the first 24 h of cold challenge. (**B**) Body temperature in mice during the seven-day cold challenge. Twelve-week-old C57BL/6J mice were subjected to a surgical or sham bilateral SNS denervation in iBAT and were then challenged with a seven-day cold exposure at 5 °C. All data are expressed as mean ± SEM; n = 7–8; * *p* < 0.05 vs. sham.

We next challenged the denervated and sham control mice with a 5 °C cold in a seven-day time course. As expected, body temperature of denervated mice dropped to a significantly lower level than that of sham controls during the first 24 h of cold exposure (Figure 3A). Body temperature of denervated mice then gradually recovered along the course of cold exposure, which eventually reached a level comparable to that of the sham control mice on day six (Figure 3B). These data clearly depict a temperature recovery process, which motivated us to investigate the underlying factors responsible for maintaining the body temperature in the absence of iBAT SNS innervation.

**Figure 4:**
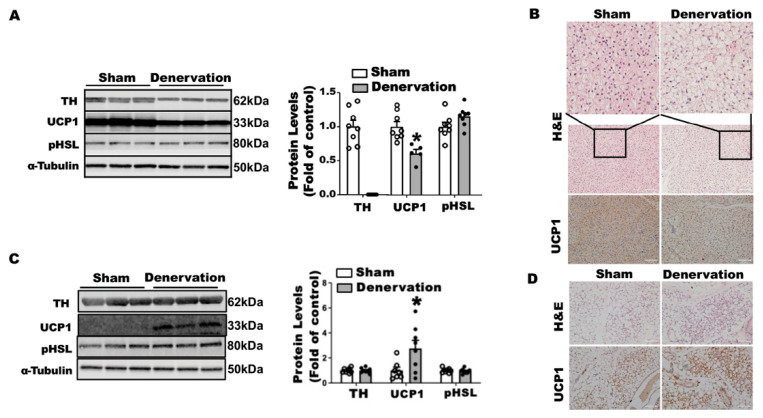
Surgical iBAT SNS denervation reduces TH and UCP1 protein levels in iBAT while increasing beiging in iWAT of mice challenged with an acute 16-h cold exposure. (**A**) Immunoblots of TH, UCP1 and pHSL protein in iBAT. (**B**) H&E images and immunohistochemical staining of UCP1 in iBAT. (**C**) Immunoblots of TH, UCP1 and pHSL protein in iWAT. (**D**) H&E images and immunohistochemical staining of UCP1 in iWAT. Twelve-week-old C57BL/6J male mice were subjected to a surgical or sham bilateral SNS denervation in iBAT and were then challenged with an overnight (16 h) exposure at 5 °C. All data are expressed as mean ± SEM; n = 5–8; * *p* < 0.05 vs. sham.

To determine what drove body temperature lower in the denervated mice in the first 16 h of cold exposure, we characterized the iBAT and iWAT of these animals. There were no differences in body weight between the denervated mice and sham controls after a 16-h cold challenge (Supplementary Figure S2A), nor were there any differences in iBAT and other fat pad weight except for a lighter color observed in denervated iBAT (Supplementary Figure S2B,C). As expected, surgical iBAT SNS denervation completely depleted TH protein levels in iBAT (Figure 4A). The absence of SNS innervation resulted in an 40% reduction in UCP1 protein levels without changes in phospho-HSL in iBAT (Figure 4A). Immunohistochemical analysis revealed larger brown adipocytes and less UCP1 staining in denervated iBAT (Figure 4B). In contrast, iBAT SNS denervation markedly induced UCP1 protein in iWAT without altering TH protein and pHSL levels (Figure 4C). Further immunohistochemical analysis showed more UCP1-positive multilocular adipocytes in iWAT of denervated mice (Figure 4D), suggesting an induction of beiging. These data imply that the induction of beige adipocytes is not sufficient to compensate for the iBAT dysfunction by the loss of sympathetic innervation, as denervated mice still experienced a lower body temperature in the early stage of acute cold exposure.

**Figure 5:**
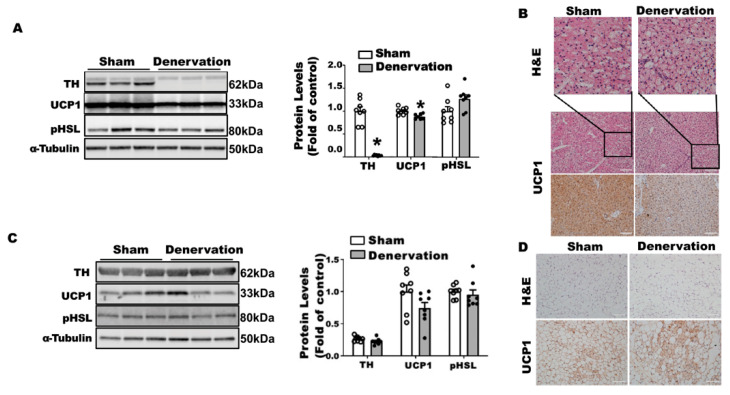
Surgical iBAT SNS denervation reduces TH and UCP1 protein levels in iBAT while slightly increasing beiging in iWAT of mice challenged with a chronic seven-day cold exposure. (**A**) Immunoblots of TH, UCP1 and pHSL protein in iBAT. (**B**) H&E images and immunohistochemical staining of UCP1 in iBAT. (**C**) Immunoblots of TH, UCP1 and pHSL protein in iWAT. (**D**) H&E images and immunohistochemical staining of UCP1 in iWAT. Twelve-week-old C57BL/6J male mice were subjected to a surgical or sham bilateral SNS denervation in iBAT and were then challenged with a seven-day cold exposure at 5 °C. All data are expressed as mean ± SEM; n = 7–8; * *p* < 0.05 vs. sham.

To determine what drove the body temperature recovery in denervated mice during prolonged cold exposure, we next examined iBAT and iWAT of the denervated mice after a seven-day cold exposure. There was no difference in body weight between denervated mice and sham controls after the seven-day cold challenge (Supplementary Figure S3A). There was also no difference in various white fat pad weight between the two groups; however, denervated mice had a smaller iBAT (Supplementary Figure S3B,C). TH protein was barely detectable in denervated iBAT (Figure 5A), suggesting a successful denervation. Up to 87.9% of UCP1 protein remained in denervated iBAT after the seven-day cold exposure (Figure 5A). This was confirmed by immunohistochemical analysis showing less UCP1 staining and larger brown adipocytes in denervated iBAT (Figure 5B). Unlike what was shown in iWAT after overnight cold, there was no difference in UCP1, pHSL and TH protein levels in iWAT of denervated mice (Figure 5C), although there was a slight increase in beige cell formation assessed by immunohistochemistry (Figure 5D). These data suggest that beiging in iWAT per se might not be a major driving force for the body temperature recovery in denervated mice during prolonged cold exposure.

**Figure 6:**
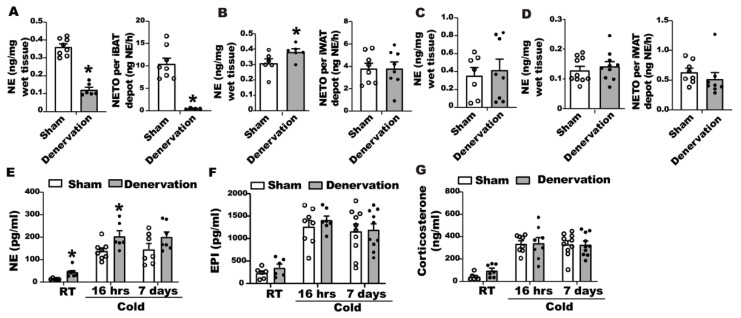
Assessment of catecholamine and glucocorticoids levels in iBAT, iWAT and blood of denervated and sham-operated mice during cold exposure. (**A**) NE content and NETO in iBAT of denervated and sham-operated mice challenged with a 16-h cold exposure. (**B**) NE content and NETO in iWAT of denervated and sham-operated mice challenged with a 16-h cold exposure. (**C**) NE content in iBAT of denervated and sham-operated mice challenged with a seven-day cold exposure. (**D**) NE content and NETO in iWAT of denervated and sham-operated mice challenged with a seven-day cold exposure. (**E**) Circulating NE levels in denervated and sham-operated mice during the seven-day cold challenge. (**F**) Circulating epinephrine levels in denervated and sham-operated mice during the seven-day cold challenge. (**G**) Circulating corticosterone levels in denervated and sham-operated mice during the seven-day cold challenge. All data are expressed as mean ± SEM; n = 7–10; * *p* < 0.05 vs. sham.

The catecholamines released from the sympathetic nerve terminals in adipose tissue during the cold exposure are the driving force for activation of brown/beige thermogenesis [23,24]. To gain a better understanding of body temperature change in denervated mice in response to cold, we assessed norepinephrine tones in iBAT and iWAT of denervated and sham-operated mice during the seven-day cold exposure time course. After a 16-h cold exposure, denervated iBAT still contained a sizable amount of basal NE (Figure 6A, left panel), albeit lower than sham controls. However, NETO remained extremely low in denervated iBAT (Figure 6A, right panel); whereas iWAT of denervated mice had increased basal NE content (Figure 6B left panel) without changes of NETO (Figure 6B right panel). The increased basal NE content in WAT might be responsible for enhanced beiging in the denervated mice.

After a seven-day cold challenge, there was no difference in basal NE content between denervated and sham-operated iBAT (Figure 6C). However, we were unable to calculate NETO in iBAT from seven-day cold exposed mice, as the NE content in iBAT of both denervated and sham-operated mice was undetectable after AMPT injection. There was also no difference in basal NE contents and NETO of iWAT between denervated mice and controls after a seven-day cold exposure (Figure 6D).

We also measured circulating catecholamine and glucocorticoids levels in denervated and sham-operated mice during the course of seven-day cold exposure. We found that cold exposure significantly increased circulating catecholamines, including NE and epinephrine (Figure 6E,F). Interestingly, the NE level in the blood circulation was higher in denervated mice than that of sham controls when mice were housed at room temperature or after a 16-h cold exposure, and tended to be higher in denervated mice than that of the sham control group after a seven-day cold exposure (Figure 6E). There was no difference in circulating epinephrine levels between the two groups (Figure 6F). In addition, cold exposure also increased circulating corticosterone levels in both denervated and sham-operated mice; however, there was no difference in circulating corticosterone levels between the two groups (Figure 6G).

**Figure 7:**
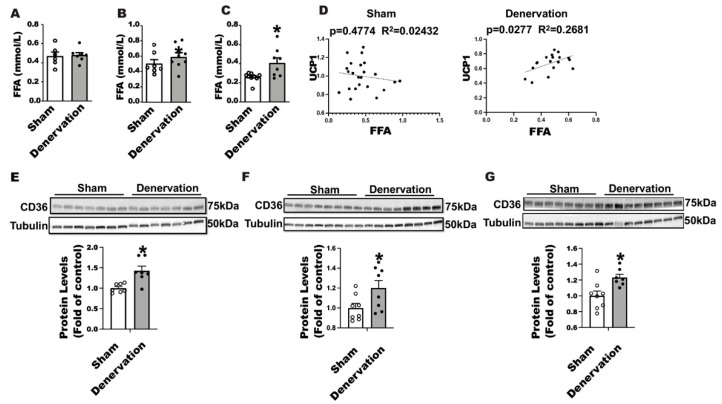
Circulating FFA levels are positively correlated with UCP1 protein contents in iBAT of denervated mice. (**A–C**) Circulating fatty acid levels in denervated and sham-operated mice housed at room temperature (**A**), challenged with a 16-h cold exposure (**B**), and challenged with a seven-day cold exposure (**C**). (**D**) Circulating FFA levels are positively correlated with UCP1 protein content in iBAT of denervated mice (right panel) but not sham mice (left panel). (**E–G**) Immunoblotting of CD36 protein in iBAT of denervated mice and sham controls housed at room temperature (**E**), challenged with an overnight (**F**) and seven -day cold (**G**). All data are expressed as mean ± SEM; n = 7–8; * *p* < 0.05 vs. sham.

Fatty acids promote brown fat thermogenesis by serving as a fuel and a UCP1 activator [23,25]. We thus measured circulating free fatty acid levels in denervated and sham-operated mice along the course of 7-day cold exposure. There was no difference in circulating fatty acid levels between denervated mice and sham controls either housed at room temperature or challenged with an overnight cold (Figure 7A,B); however, the circulating fatty acid level was significantly increased in denervated mice after a seven-day cold exposure (Figure 7C). Interestingly, we discovered a strong positive correlation between circulating FFA levels and UCP1 protein contents in iBAT of denervated mice (Figure 7D right panel) but not sham mice (Figure 7D left panel), suggesting an important role for FFA in maintaining iBAT UCP1 protein in the absence of SNS innervation. FFA influx into brown fat relies on the fatty acid transport protein CD36. Immunoblotting analysis revealed an up-regulation of CD36 protein levels in iBAT of denervated mice compared to the sham controls housed at room temperature (Figure 7E), challenged with an overnight (Figure 7F) or seven-day cold (Figure 7G), suggesting an active transport of fatty acid into brown fat in the absence of SNS innervation.

**Figure 8:**
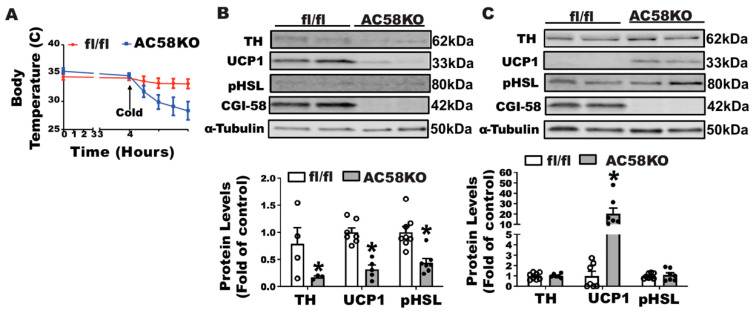
Fatty acids partially contribute to the maintenance of body temperature of denervated mice. (**A**) Body temperature of AC58KO mice with iBAT SNS denervation or sham operation challenged with an acute cold exposure at 5 °C in the absence of food. Food was removed 4 h before the initiation of cold exposure and remained absent during the cold exposure. (**B**) Immunoblots of TH, UCP1, pHSL, and CGI-58 protein in iBAT of AC58KO mice and fl/fl littermate controls challenged with an acute cold exposure. (**C**) Immunoblots of TH, UCP1, pHSL and CGI-58 protein in iWAT of AC58KO mice and fl/fl littermate controls challenged with an acute cold exposure. All data are expressed as mean ± SEM; n = 4–8; * *p* < 0.05 vs. sham.

To test the importance of fatty acids in maintaining body temperature and UCP1 expression in mice without sympathetic innervation in iBAT, we characterized cold-induced thermogenesis of a genetic mouse model (AC58KO) deficient in adipocyte Comparative Gene Identification-58 (CGI-58), a coactivator of Adipose Triglyceride Lipase (ATGL) required for intracellular lipid droplet lipolysis [21,26]. We first challenged AC58KO mice with a 5 °C cold. When deprived of food, which restricted mice from diet-derived fatty acids during the cold exposure, AC58KO mice exhibited a sharp decline of temperature, which was substantially rescued by a gavage of olive oil (Supplementary Figure S4), suggesting the key role of fatty acids in maintaining thermogenesis in these mice. We then conducted an iBAT SNS denervation in AC58KO mice. AC58KO mice and their fl/fl controls received a surgical denervation of iBAT SNS and were then challenged with a cold exposure at 5 °C, during which food was removed to avoid diet-derived fatty acids. The AC58KO mice with iBAT denervation were extremely cold-sensitive, evidenced by a significant decline of body temperature 4 h after the beginning of the cold challenge (Figure 8A), suggesting a severe cold intolerance. Characterization of iBAT revealed a marked down-regulation of UCP1 protein and phospho-HSL in AC58KO mice (Figure 8B). By contrast, UCP1 protein was significantly up-regulated in iWAT of AC58KO mice, although no change was observed in phospho-HSL (Figure 8C).

## 4. Discussion

The premise of this study was derived from our prior observations on the hamsters with iBAT SNS denervation [20]. As much as the importance of SNS innervation in BAT thermogenesis, hamsters lacking iBAT SNS innervation with chemical SNS denervation were not only viable but also displayed a normal core temperature when challenged with cold at 4 °C. These animals benefited from an induction of beige adipocytes due to enhanced SNS innervation into iWAT, which offsets the loss of iBAT thermogenesis [20]. Similar results were also observed in mice with iBAT chemical SNS denervation [19]. One caveat is that iBAT SNS denervation in these studies is a partial denervation achieved by microinjection of 6OHDA [19,20]. It is not clear whether the remaining sympathetic innervation may be responsible for the preservation of certain BAT thermogenesis that is sufficient to maintain a proper body temperature. In this present study, a surgical denervation was employed to achieve complete denervation of SNS in iBAT. Mice with surgical denervation of SNS in brown fat were also viable during cold exposure and were able to recover body temperature to the level comparable to that of control mice.

We want to first highlight two observations that link the dynamic changes of iBAT UCP1 protein and NE contents to the fluctuated body temperature of denervated mice during the cold exposure. For one, the pattern of more reduction of UCP1 protein (40%) in denervated iBAT of 16 h vs. lesser reduction of UCP1 (12%) in denervated iBAT of seven-day cold mirrors the dynamic change of body temperature: the body temperature of denervated mice falls at the early stage of cold exposure and gradually recovers along the course of cold exposure, which eventually reaches to a level comparable to that of sham control mice on day six. This observation highlights the importance of brown fat UCP1 in maintaining body temperature. On the other hand, the change of denervated iBAT UCP1 protein from 16 h to seven-day also parallels the pattern of NE contents in denervated iBAT along the course of cold exposure: lower iBAT NE content in 16 h cold exposure when body temperature declines and higher iBAT NE content in seven-day cold when body temperature recovers. This also underscores the importance of brown fat NE in maintaining brown fat UCP1 protein and body temperature.

Severing SNS surgically indeed achieved a complete denervation in iBAT, evident by complete loss of TH protein, barely detectable basal NE content and NETO in iBAT. However, in the absence of SNS-derived NE, the denervated iBAT still partially recovered basal NE, especially in mice with the 16-h and seven-day cold challenge, which may be replenished by circulating NE that displays a significant increase during the cold challenge on day 1 and a tendency of increase on day seven. Although the circulating NE only exhibits a trend of increase on day seven, iBAT NE contents are similar between denervated mice and the sham controls, suggesting that a dynamic replenishment of NE into denervated iBAT may drain circulating NE to a lower level. With this in mind, future studies are warranted to examine the pathways underlying NE metabolism in fat tissues such as recently identified NE transporters in sympathetic neuron-associated macrophages and adipocytes [27,28]. On the other hand, other circulating factors such as those derived from hepatokines (e.g., FGF21) may also contribute to the cold tolerance in denervated mice [6].

The circulating NE may be the major source for the increased basal NE in iWAT of the denervated mice with overnight cold exposure because the SNS innervation into iWAT of the denervated mice remains at the same level as that of the sham controls evident by unchanged NETO and TH protein. The increased NE in circulation is presumably derived from adrenal medulla in response to the stress such as cold and loss of iBAT innervation. However, future studies involving adrenalectomy would be required to confirm whether circulating NE compensates for the loss of SNS-derived NE in sustaining thermogenesis during cold exposure. Although alternatively activated macrophages (M2) also have been shown to produce catecholamines that promote BAT thermogenesis and lipolysis [29], the study has been challenged by a follow-up study indicating that alternatively activated macrophages do not contribute significantly to tissue catecholamine levels [30]. The basal NE in adipose tissue might be important for maintaining a sizable amount of UCP1 protein in iBAT, which help maintain a proper body temperature during the time course of cold.

Chemical denervation of iBAT in hamsters indeed impaired BAT thermogenesis evident by lower BAT temperature [20]. However, the loss of BAT thermogenesis was simultaneously compensated by increased WAT thermogenesis that likely resulted from newly acquired beige adipocytes due to enhanced SNS outflow to WAT [20]. Hence, chemically denervated hamsters did not experience a drop in body temperature at the beginning of cold, because beige adipocyte thermogenesis seems to be a significant contributor to body temperature. This was not the case in our surgically denervated mouse model. In the current study, the surgically denervated mice experienced a decline of body temperature at the early stage of cold challenge, despite a significant induction of beiging observed in denervated mice at the beginning of cold exposure. Thus, beige adipocyte thermogenesis does not appear to be an impactful contributor to body temperature in surgically denervated mice during the acute cold exposure. For one, a significant induction of beiging occurred in denervated mice that exhibited lower temperatures at the beginning of cold, suggesting that the presence of beige adipocytes is not sufficient to prevent the decline of body temperature. On another note, the beiging effect in iWAT became less significant during the later stage of the seven-day cold exposure when the denervated mice recovered the body temperature, which is also different from our previous study in mice with chemical iBAT SNS denervation, where the induction of UCP1 protein levels and beiging in iWAT of denervated mice were still significant after a seven-day cold exposure [19]. The discrepancy between the current study and these previous studies is not clear. It might be that hamsters and mice are two different models that may have different thermoregulatory systems in response to cold. In addition, these studies employed two different approaches, chemical and surgical denervation, respectively, which may create different outcomes. For example, a chemical approach can only achieve partial denervation, which inevitably leaves some SNS in iBAT. On the other hand, a surgical approach not only severs SNS but also sensory nerves. The outcome of sensory denervation is not clear. However, we recently showed that sensory denervation in iWAT alters SNS outflow to iBAT [31], suggesting a role of fat tissue sensory nerve in mediating the coordination of BAT and WAT thermogenesis and metabolism. Future studies are required to determine the role of BAT sensory nerves in cross talk between BAT and WAT.

We believe that fatty acids play a significant role in maintaining iBAT UCP1 protein and thermogenesis during cold exposure as we observed a significant increase in circulating fatty acid levels on day seven of cold challenge when the denervated mice recovered their body temperature. In support of this, we observed a strong correlation between circulating fatty acid levels and iBAT UCP1 protein in denervated mice, suggesting the importance of fatty acids for the mice to maintain the sizable brown fat UCP1 in the absence of SNS innervation. Further, the increased expression of CD36 protein in iBAT of denervated mice indicates that circulating fatty acids are actively transported into brown adipocytes and may serve as a driving force to retain the UCP1 protein in the denervated brown fat. To further confirm the vital role of fatty acids in this process, we examined the phenotype of AC58KO mice lacking lipolytic capacity, which are extremely cold sensitive with little presence of UCP1 protein in iBAT. Fatty acids serve as not only a fuel but also as a UCP1 activator for BAT thermogenesis [23,25]. Circulating fatty acids can be derived from WAT lipolysis and chylomicron hydrolysis after a meal [25]. Mice lacking adipose lipolysis were cold sensitive only when food was absent [21]. In fact, SNS promotes BAT thermogenesis partially through WAT lipolysis-derived fatty acids. NE released from the sympathetic nerve terminals in response to cold not only activates BAT thermogenesis but also stimulates WAT lipolysis, both of which are mediated via the β3-adrenergic receptor [23,24,32,33,34]. Taken together, SNS innervation of iBAT becomes dispensable for cold-induced thermogenesis when circulating fatty acids are available.

In sum, we show that mice with surgical denervation of iBAT SNS are able to tolerate a seven-day cold challenge, with proper maintenance of UCP1 protein in iBAT and body temperature during the cold challenge. The denervated mice have a significant increase in circulating NE, which may account for the basal NE levels in the denervated iBAT, maintaining UCP1 protein. Furthermore, the denervated mice also display increased free fatty acid levels in circulation, which may be important for maintaining UCP1 expression and activity in iBAT in the absence of sympathetic innervation. Indeed, surgical denervation of mice deficient in WAT lipolysis dramatically reduces UCP1 protein and causes susceptibility to cold. We conclude that circulating fatty acids and NE are the key factors for maintaining a proper cold-induced thermogenesis in the absence of BAT sympathetic innervation.

## Data Availability

All datasets will be available upon request to the corresponding authors Liqing Yu, Hang Shi and Bingzhong Xue.

## Supplementary Materials

The following are available online at https://www.mdpi.com/article/10.3390/biom11101428/s1, Figure S1: Mice with a bilateral surgical SNS denervation in interscapular BAT (iBAT) after a 7-day recovery. (A) Body weight of denervated and sham-operated mice housed at room temperature. (B) Image of iBAT of denervated and sham-operated mice housed at room temperature. (C) Fat pad weight of denervated and sham-operated mice housed at room temperature. All data are expressed as mean ± SEM; n = 6–7. Figure S2: Mice with a bilateral surgical SNS denervation in iBAT after a 16-hour cold exposure. (A) Body weight of denervated and sham-operated mice after a 16-hour cold exposure. (B) Image of iBAT of denervated and sham-operated mice after a 16-hour cold exposure. (C) Fat pad weight of denervated and sham-operated mice after a 16-hour cold exposure. All data are expressed as mean ± SEM; n = 8. Figure S3: Mice with a bilateral surgical SNS denervation in iBAT after a 7-day cold exposure. (A) Body weight of denervated and sham-operated mice after a 7-day cold exposure. (B) Image of iBAT of denervated and sham-operated mice after a 7-day cold exposure. (C) Fat pad weight of denervated and sham-operated mice after a 7-day cold exposure. All data are expressed as mean ± SEM; n = 8. * *p* < 0.05 vs. sham. Figure S4. Olive oil gavage substantially rescues the temperature of AC58KO mice challenged with cold in the absence of food. 4 hours before the cold exposure, food was removed from the cage and followed by olive oil (200 µl, water as a control) gavage 30 min before the cold exposure. All data are expressed as mean ± SEM; n = 4. * *p* < 0.05 vs. water.
