# Supplementary material for: Fatty Acids Rescue the Thermogenic Function of Sympathetically Denervated Brown Fat
Source: Biomolecules. 2021 Sep 29;11(10):1428. doi: 10.3390/biom11101428 (PMC8533276; doi:10.3390/biom11101428)

Supplemental Figure legends

Supplemental figure S1. Mice with a bilateral surgical SNS denervation in interscapular BAT (iBAT) after a 7-day recovery. (A) Body weight of denervated and sham-operated mice housed at room temperature. (B) Image of iBAT of denervated and sham-operated mice housed at room temperature. (C) Fat pad weight of denervated and sham-operated mice housed at room temperature. All data are expressed as mean ± SEM; n = 6-7.

Supplemental figure S2. Mice with a bilateral surgical SNS denervation in iBAT after a 16-hour cold exposure. (A) Body weight of denervated and sham-operated mice after a 16-hour cold exposure. (B) Image of iBAT of denervated and sham-operated mice after a 16-hour cold exposure. (C) Fat pad weight of denervated and sham-operated mice after a 16-hour cold exposure. All data are expressed as mean ± SEM; n = 8.

Supplemental figure S3. Mice with a bilateral surgical SNS denervation in iBAT after a 7-day cold exposure. (A) Body weight of denervated and sham-operated mice after a 7-day cold exposure. (B) Image of iBAT of denervated and sham-operated mice after a 7-day cold exposure. (C) Fat pad weight of denervated and sham-operated mice after a 7-day cold exposure. All data are expressed as mean ± SEM; n = 8. *$p<0.05$ vs. sham.

Supplemental figure S4. Olive oil gavage substantially rescues the temperature of AC58KO mice challenged with cold in the absence of food. 4 hours before the cold exposure, food was removed from the cage and followed by olive oil (200 μl, water as a control) gavage 30 min before the cold exposure. All data are expressed as mean ± SEM; n = 4. *$p<0.05$ vs. water.

Supplemental figure S1

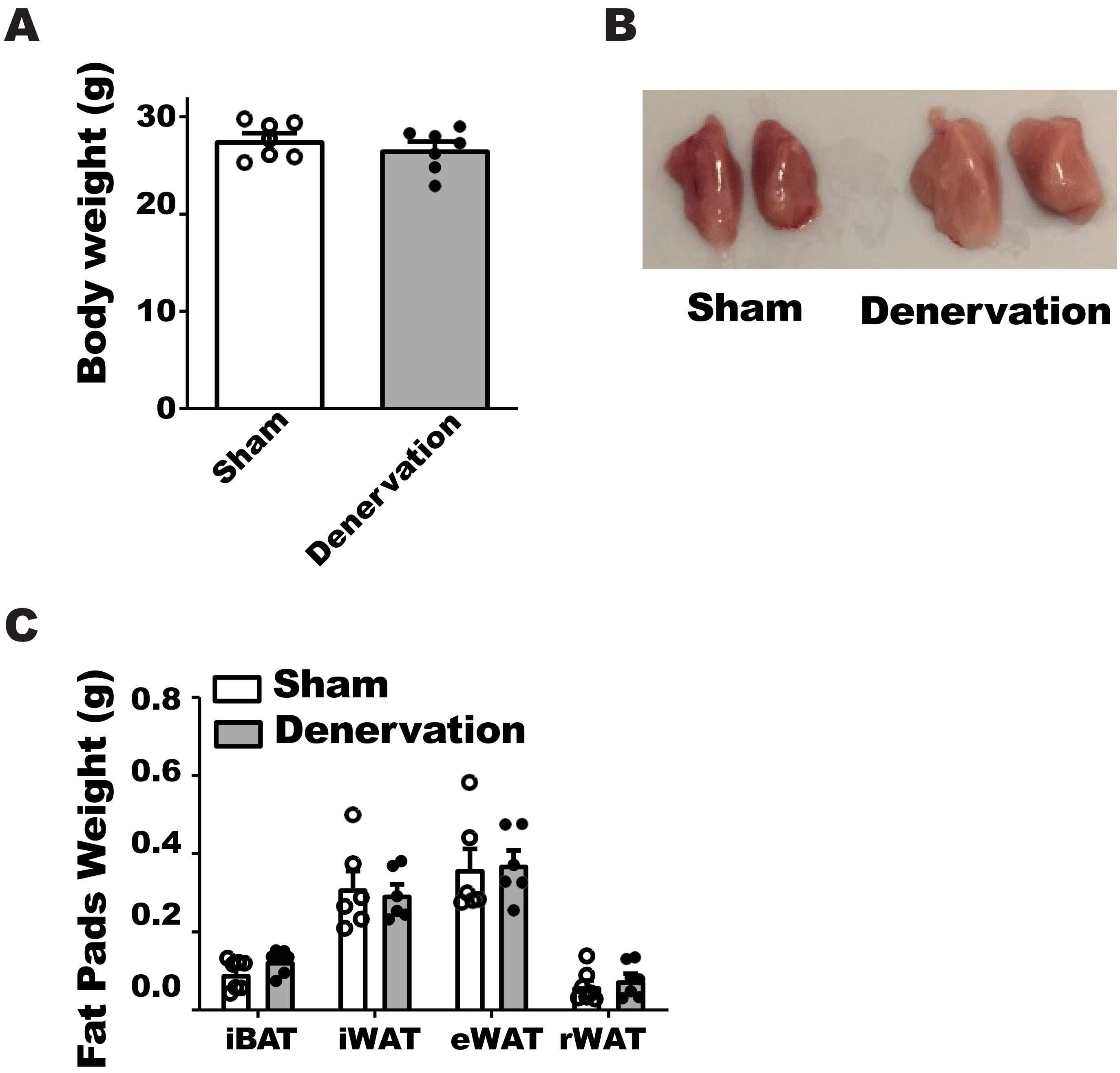

Supplemental figure S2

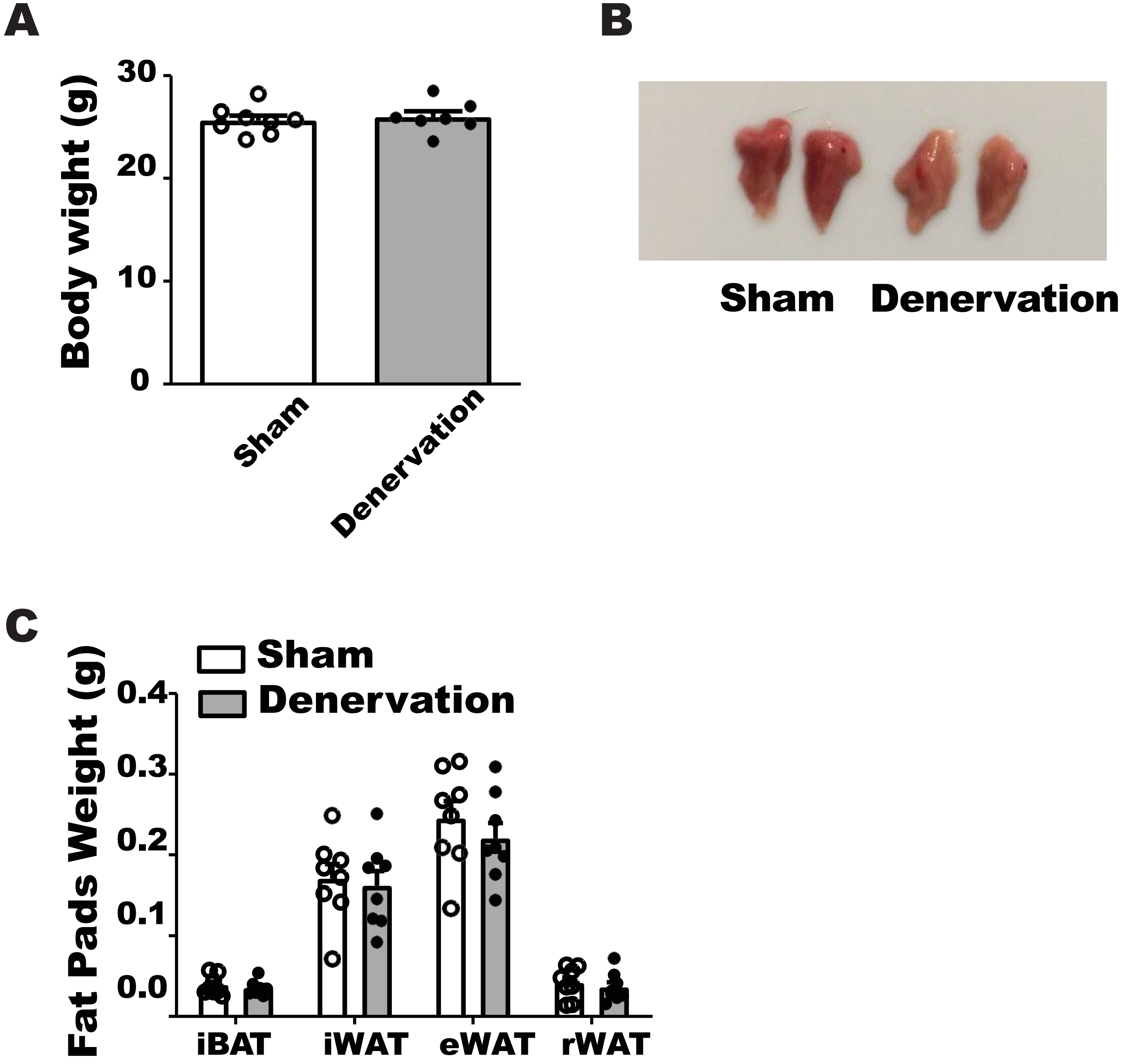

Supplemental figure S3

A

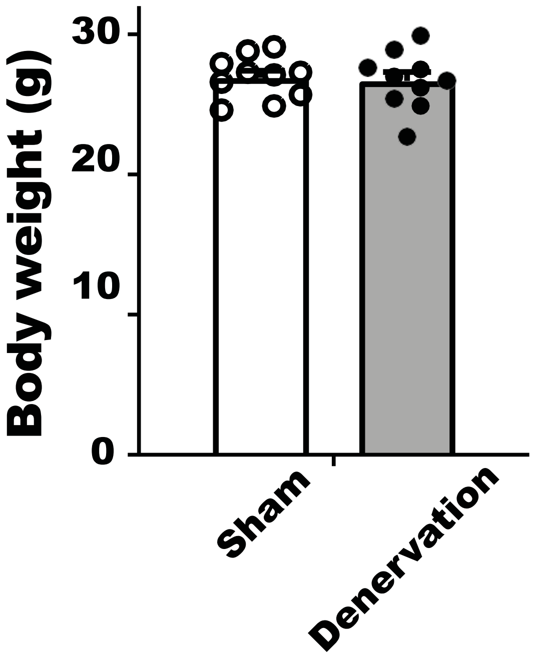

B

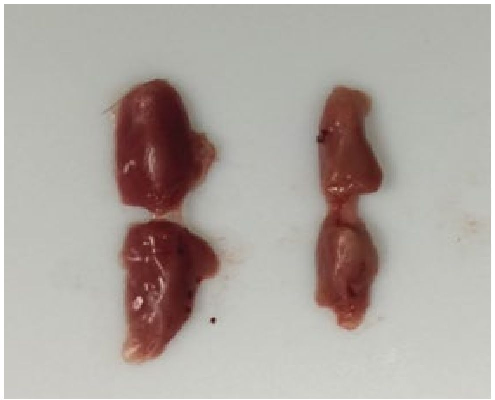

Sham Denervation

C

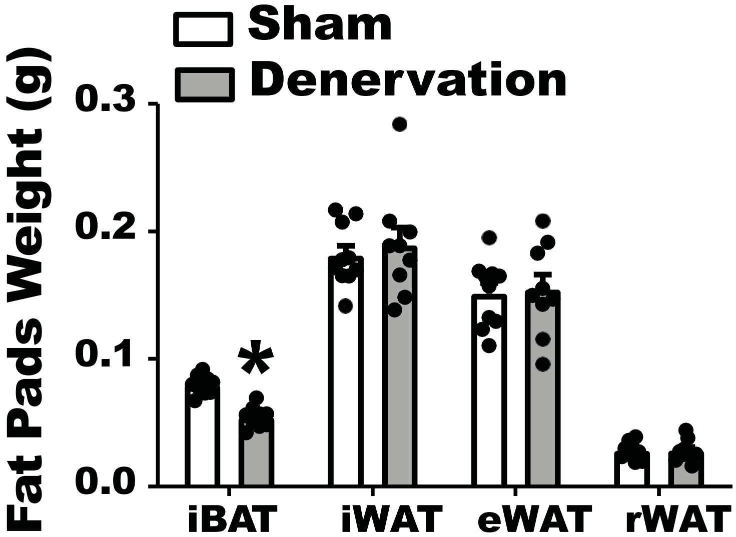

Supplemental figure S4

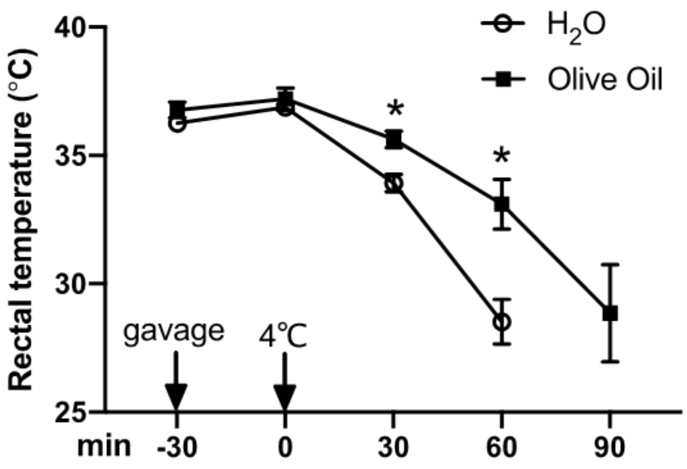

Supplement: Supplementary file 1 [file biomolecules-11-01428-s001.zip › biomolecules-1356603-supplementary.pdf]
